# Biflavonoids: Important Contributions to the Health Benefits of Ginkgo (*Ginkgo biloba* L.)

**DOI:** 10.3390/plants11101381

**Published:** 2022-05-23

**Authors:** Dunja Šamec, Erna Karalija, Sabina Dahija, Sherif T. S. Hassan

**Affiliations:** 1 Department of Food Technology, University North, Trga Dr. Žarka Dolinara 1, 48000 Koprivnica, Croatia; 2 Department for Biology, Faculty of Science, University of Sarajevo, Zmaja od Bosne 33-35, 71000 Sarajevo, Bosnia and Herzegovina; erna.k@pmf.unsa.ba (E.K.); sabina_dudevic@yahoo.com (S.D.); 3 Department of Applied Ecology, Faculty of Environmental Sciences, Czech University of Life Sciences Prague, Kamýcká 129, 165 00 Prague, Czech Republic; sherif.hassan@seznam.cz

**Keywords:** *Ginkgo biloba* L., biflavonoids, ginkgetin, isoginkgetin, bilobetin, sciadopitysin, amentoflavone, bioactive compounds

## Abstract

Ginkgo (*Ginkgo biloba* L.) is one of the most distinctive plants, characterized by excellent resistance to various environmental conditions. It is used as an ornamental plant and is recognized as a medicinal plant in both traditional and Western medicine. Its bioactive potential is associated with the presence of flavonoids and terpene trilactones, but many other compounds may also have synergistic effects. Flavonoid dimers—biflavonoids—are important constituents of ginkgophytopharmaceuticals. Currently, the presence of 13 biflavonoids has been reported in ginkgo, of which amentoflavone, bilobetin, sciadopitysin, ginkgetin and isoginkgetin are the most common. Their role in plants remains unknown, but their bioactivity and potential role in the management of human health are better investigated. In this review, we have provided an overview of the chemistry, diversity and biological factors that influence the presence of biflavonoids in ginkgo, as well as their bioactive and health-related properties. We have focused on their antioxidant, anticancer, antiviral, antibacterial, antifungal and anti-inflammatory activities as well as their potential role in the treatment of cardiovascular, metabolic and neurodegenerative diseases. We also highlighted their potential toxicity and pointed out further research directions.

## 1. Introduction

**Figure 1:**
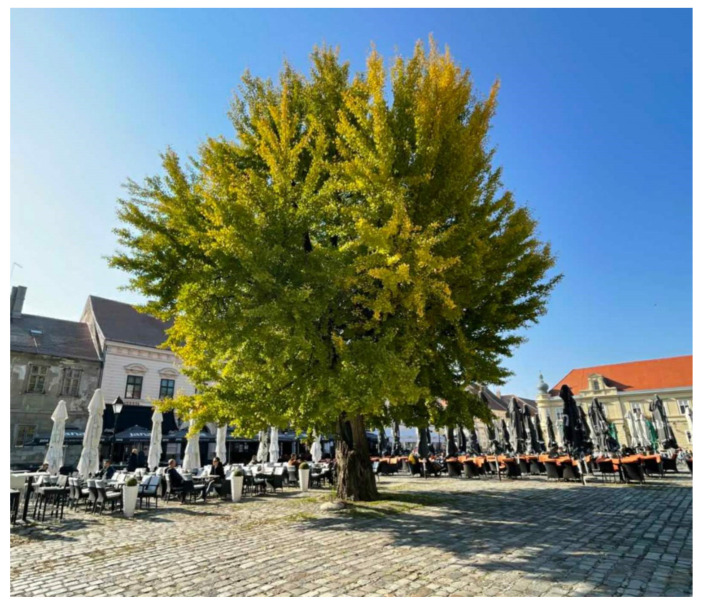
Approximately 200-year-old ginkgo tree in the old part of the city of Osijek, Croatia (photo taken in October 2021).

*Ginkgo biloba* L., known as Maidenhair tree or ginkgo, is the only living dioecious species in the family Ginkgoaceae [1,2,3]. The species grows in small natural populations in southeastern China but is cultivated throughout China and the world [4,5]. The morphology of ginkgo is very conservative, and this species exhibits remarkable genetic stability [6,7]. The ginkgo species is characterized by a large genome and high tolerance to stress factors, including abiotic and biotic stress and dioecious reproduction [8]. High tolerance to stress is demonstrated not only by the fact that this tree is a living fossil but also through its survival of the Hiroshima atomic bomb, with miraculous recovery within 1–2 km of the blast. In horticulture, male trees are predominantly used due to the unpleasant odor from fruit that female trees produce [9]. The unpleasant odor of ginkgo seeds develops only when they are fully mature and is the result of two volatile compounds, butanoic and hexanoic acids, localized in the sarcotesta [10]. The species was introduced to other Asian and European countries, mainly for ornamental and greening purposes [11,12]. Today, ginkgo trees, often more than 200 years old, can be found as ornamental plants in many European cities (Figure 1).

In traditional Chinese medicine, leaves and fruits have been used for several centuries with documented use dating back from 1280 to 1368 AD and the use of ginkgo nuts mentioned as early as the Yuan Dynasty [13]. For more than 500 years, various medicinal properties have been attributed to the nuts, including melioration of asthma, cough, and bladder infections, while leaves have been used mainly to treat skin infections and pulmonary dysfunction (reviewed by [14,15]). Although it is well used in Chinese medicine, the true value of this 200-million-year-old plant species was recognized only 40 years ago. The best-known properties of ginkgo are its beneficial effects on cognitive complaints, which became known through the commercial sale of a standardized extract formulation developed in Germany in 1965. However, commercial use under the name EGb761 was only in 1974 in France and is now used worldwide [15]. In addition to its medicinal properties, the fruits of ginkgo are used for the preparation of jams, especially for weddings and other celebrations. Roasted seeds are a delicacy in many East Asian countries such as Japan, China, Malaysia, and Korea [16]. 

Due to its long use in traditional medicine, ginkgo is considered a natural reservoir of molecules with health-promoting potential. The health-promoting properties of ginkgo are associated with the presence of specialized metabolites such as flavonoids, terpenoids and trilactones [17]. According to recent data, more than a hundred different flavonoid structures have been detected in ginkgo [18]. They exist as aglycones, glycosides, or dimeric forms called biflavonoids. Biflavonoids are dimers of flavone–flavone, flavone–flavonone, flavonone–flavonone subunits, and in rare cases, dimers of chalcones and isoflavones. As summarized by He et al. [19], a total of 592 biflavonoids are widely distributed in angiosperms, ferns, gymnosperms, and bryophytes, with most of them found in angiosperms. The vast majority of biflavonoids are from Clusiaceae, Thymelaeaceae, Ochnaceae, and Selaginellaceae, which account for about 50% of biflavonoids in all families [19]. Their presence is frequently reported in plants used in traditional and modern medicine, indicating their potential health benefits. Therefore, it is not surprising that biflavonoids have become the focus of scientific attention in recent years due to their pharmacological activities, including anti-inflammatory, antioxidant, antibacterial, antiviral, antidiabetic, antitumor, and cytotoxic properties, and may be used to treat Alzheimer’s and Parkinson’s disease [19]. To date, 13 different biflavonoids have been reported, of which ginkgetin, isoginkgetin, amentoflavone, bilobetin and sciadopitysin are the predominant biflavonoids [18]. 

Although biflavonoids are recognized as compounds with potent health-promoting properties, they have somehow been neglected in studies dealing with flavonoids in ginkgo. However, considering the use of ginkgo in traditional and modern medicine and the biological properties of biflavonoids, they may play an important role in the biological activity of ginkgo. Thus, in this review, we summarize data on ginkgo biflavonoids, their occurrence in various plant tissues, their biological activity, pharmacological potential, and safety to highlight this important class of natural products.

## 2. Botanical Characteristic of Ginkgo

**Figure 2:**
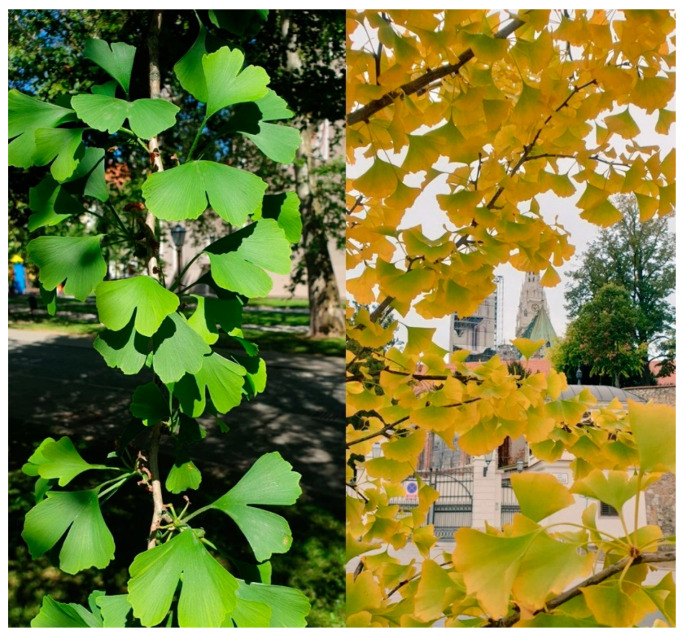
The color of young (**left**) and mature (**right**) *G. biloba* leaves.

Mature ginkgo trees typically reach a height of 20 to 40 m and a trunk diameter of one to four meters [20]. They form two types of shoots: long shoots with widely spaced leaves and axillary buds, and short shoots with clustered leaves with no internodes nor axillary buds. The leaves have long stalks and a characteristic fan-shape with dichotomously branched veins. They are light green in color and turn golden yellow in the fall (Figure 2). The tree bark is grey in color, smoots in older trees with longitudinal cracks turning the bark color to brown. The flowers are dioecious and grow only on short shoots. 

The male catkins appear before the leaves and pollination occurs from early April to late May by the wind. The seeds produced are large (20–30 mm × 16–24 mm), with a thick seed coat containing embryos embedded in the tissue of the female gametophyte [21]. The structure of the seed coat is complex and consist of several layers: a soft, fleshy outer layer (the sarcotesta); a hard, stony middle layer; and a thin, membranous inner layer. Sex maturity in ginkgo is reached only after 20 years of age [9]. Vegetative reproduction is possible through embedded buds—lignotubers (basal chichi)—which are formed at the base of the main stem [22].

## 3. Phytochemicals in Ginkgo

As mentioned above, the phytochemicals of ginkgo have attracted the attention of scientists for several decades due to their long-standing use in traditional medicine. About 15 years ago, Singh et al. [23] categorized the phytochemicals found in ginkgo into the groups of terpenoids, polyphenols, allylphenols, organic acids, carbohydrates, fatty acids and lipids, inorganic salts and amino acids. In recent years, some new types of compounds such as lignans and new terpenoids have been identified [18]. Recently, Liu et al. [18] summarized all the data on phytochemicals in ginkgo published from 2015 to 2020 and found that 184 papers focused on phytochemicals found in ginkgo leaves during this period alone. In their article, Liu et al. [18] divided the phytochemicals associated with the health benefits on ginkgo into the group of flavonoids, terpenoids, alkylphenols and alkylphenolic acids, carboxylic acids, lignans, proanthocyanidins, polyprenols and polysaccharides. Characteristic of ginkgo are terpene trilactones such as ginkgolides and bilobalide, which are unique to ginkgo and are the only natural products that have a t-butyl group in their structure [23]. *N*-glycosides such as *N*-β-d-glucopyranosyl-1H-indole-3-acetic acid conjugates, *N*-[2-(1-β-d-glucopyranosyl)-1H-indol-3-yl) acetyl]-l-glutamic acid and *N*-[2-(1-β-d-glucopyranosyl)-1H-indol-3-yl)acetyl]-l-aspartic acid, have also been isolated from ginkgo parts, which are rarely found in nature as natural products [24]. Ginkgo seeds contain toxic compounds such as ginkgotoxin, ginkgotoxin-5-glucoside, ginkgolic acid, amygdalin-derived hydrocyanic acid and allergic proteins, which restricts daily consumption [25].

The standardized extract preparation of ginkgo leaves usually contains 24% flavonoids, 6% terpene trilactones (TTLs), and less than 5 ppm ginkgolic acid [17]. Flavonoids are a group of specialized plant metabolites that are important for plant–environment interactions [26,27] but have also been associated with numerous human health benefits [28,29,30,31,32]. Flavonoids are composed of a 15-carbon skeleton and consist of two benzene rings (ring A and ring B) connected by a 3-carbon linking linkage. Therefore, they are represented as C_6_-C_3_-C_6_ compounds. In many flavonoids, the linking chain also forms a heterocyclic pyran or pyrone ring (ring C). In the absence of this third ring system (ring C), flavonoids are referred to as chalcones and usually serve as precursors to the various flavonoid classes. In plants, flavonoids occur in two different forms, including free aglycones and glycoside-bound forms. Based on their structural differences, flavonoids are dividated into several groups- flavanones, flavanols, flavones, and flavonols. In addition, flavonoids include isoflavones, prenylflavonoids, glycosidoesters, flavonolignans, aurones, chalcones and biflavonoids. 

According to the review article by Liu et al. [18], 110 flavonoids with unique structures have been detected in ginkgo, including flavonol and its glycosides, flavone and its glycosides, flavanone and its glycosides, isoflavone and its glycosides, flavan-3-ols, biflavonoids, and biginkgosides. Most of them, 52 reported structures, belong to the flavonol glycosides. The predominant flavonol aglycones in the glycosides are quercetin, kaempferol, and isorhamnetin, but also syringetin, myricetin, laricitrin, myricetin 3’,4’-dimethyl-ether, and patuletin were also found [18]. According to Wang et al. [33], the predominant compounds in ginkgo leaves during the different harvesting periods were flavonol glycosides, especially kaempferol-3-*O*-rutinoside and isorhamnetin-3-*O*-rutinoside. Other glycosides present in ginkgo are derivatives of flavones (mainly apigenin and luteolin), flavanones, isoflavones and flavan-3-ols [18]. In recent years, the presence of biginkgosides, flavonol glycoside cyclodimers, has been demonstrated in ginkgo [34]. To date, nine different structures of biginkosides have been described [34]. Several dimeric flavonoid structures have also been found in ginkgo which may be important contributors to the health benefits of ginkgo.

## 4. Biflavonoids in Ginkgo

In nature, in certain plant species, flavonoid dimerization may occur. Flavonoid dimers, known as biflavonoids, consist of two identical or non-identical flavonoid units joined in a symmetrical or asymmetrical manner, by an alkyl or an alkoxy-based linker of varying length. Most representative compounds of this class are formed by dimers of flavone–flavone, flavone–flavonone, flavonone–flavonone subunits, as well as dimers of chalcones and isoflavones, in rare cases. Their role in plants remains unknown. Based on in vitro biological activity and localization in the leaves, they might be involved in plant protection from pests and predators, act as growth regulators or protect leaves from UV radiation [35,36], but further experimental data should be collected to support these statements. 

### 4.1. Chemistry and Diversity of Ginkgo Biflavonoids

The first biflavonoid, ginkgentin, was isolated from ginkgo in 1929. as a yellow pigment [37] and since then, numerous biflavonoid compounds have been isolated from various plants. He et al. [19] reported that more than 592 biflavonoids have been structurally elucidated, but the actual number is probably even higher. In ginkgo, 13 different biflavonoids have been described so far [18]. Their structures are presented in a Table 1 and Table 2. 

The most commonly reported biflavonoids in ginkgo are ginkgetin, isoginkgetin, amentoflavone, bilobetin and sciadopitysin, while the presence of sesquojaflavone, podocarpusflavone A, and 5’-metoxybilobetin has also been reported [18,33]. Amentoflavone is a dimer of two apigenins with six hydroxyl groups that can be easily replaced by a methoxyl group. Therefore, other biflavonoids can also be considered as derivatives of amentoflavone. In ginkgo sequoiaflavone (7-*O*-methylamentoflavone), bilobetin (4′-*O*-methylamentoflavone) and podocarpusflavone A (4′′′-*O*-methylamentoflavone) were found to be the natural derivatives with a single methoxyl group. Ginkgetin (7,4′-di-*O*-methylamentoflavone), isoginkgetin (4′,4′′′-di-*O*-methylamentoflavone) and 5’-methoxybilobetin have two methoxyl groups, while three methoxyl groups are present in sciadopitysin (7,4′,4′′′-tri-*O*-methylamentoflavone). Similar to monomeric flavonoids, biflavonoids tent to form glycosides. To date, the presence of 7′′-*O-β-*d-glucosyl-ginkgetin, 7′′-*O*-β-d-glucosyl-isoginkgetin and 7′′-*O*-β-d-glucosyl-amentoflavone has been reported in ginkgo [18]. In the structure of amentoflavone, the carbon–carbon double bonds of C2-C3 are easily hydrogenated. So far, the presence of 2,3-dihydroisoginkgetin and 2,3-dihydrosciadopitysin in ginkgo has been demonstrated [18]. Most of the ginkgo biflavonoids reported so far can be classified as a BB or flavone–flavone type [19].

As can be seen from the structures in Table 1 and Table 2, the separation and identification of biflavonoids can be challenging, because of the rather similar structure among some of them. It requires the use of modern techniques for separation and identification such as various hyphened methods, which are still expensive and not widely available. In addition, commercial availability of standards is limited to ginkgetin, isoginkgetin, amentoflavone, bilobetin and sciadopitysin, which may be the reason why these biflavonoids are the most commonly reported in ginkgo. To obtain a complete picture of the presence and metabolism of biflavonoids in ginkgo, further untargeted metabolomics experiments should be conducted to search for additional biflavonoids in ginkgo. These data would shed light on the still unknown role of biflavonoids in plant growth, development and communication with the environment. 

### 4.2. Biological Factors Which Influence the Presence of Biflavonoids in Ginkgo

The amount and presence of individual biflavonoids depend on plant part, stage of development and environmental conditions during growth. Table 3 provides examples of selected reports on the presence of biflavonoids in various ginkgo plant parts and the used extraction and identification methods. Various organic solvents such as acetone, methanol, ethanol and petroleum ether have been used for the isolation of biflavonoids [38,39,40,41,42,43,44,45,46,47,48,49]. Recent data also show that sustainable extraction technologies [43] and modern extraction techniques [42] can be useful for the extraction of biflavonoids from ginkgo. The method of choice for the separation of compounds is liquid chromatography. Because of the characteristic UV absorption, DAD detectors can be used to detect biflavonoids. However, for accurate confirmation of structures DAD detectors are often combined with mass spectrometry and/or NMR, especially when standards are not commercially available. 

Lei et al. [43] extracted four biflavonoids—bilobetin, gingketin, isoginkgetin and sciadopitysin—from the leaves of ginkgo, of which sciadopitysin was the most abundant. The same biflavonoids in leaves were reported by Beck and Stengel [40]. In addition, they performed MALDI mass spectrometric imaging on leaf section and found that all detected biflavones were located at the leaf surface and the concentration was increased on the lower leaf surface compared to the upper surface. This could be related to their biological function. Pandey et al. [44] analysed the content of amentoflavone and sciadopitysin in leaves, stems, and fruits of ginkgo trees. They detected those biflavonoids in all analysed parts, although the sciadopitysin content was about 200 times higher in all samples. The content of amentoflavone in female trees was similar in all samples, while it was higher in male leaves. Sciadopitysin content in younger female leaves was lower than in the stem, while in older plants, it was more than twice that in the stem or fruit. Li et al. [45] reported the presence of amentoflavone, bilobetin, and isoginkgetin sciadopitysin but also amentoflavone 7″-*O*-d-glucopyranoside in male flowers. Chen et al. [48] found only isoginkgetin in leaves and seed coats among the 25 compounds identified in leaves, seed coats and embryoids of ginkgo, while it was absent in embryoids. Isoginkgetin was present in leaves at much higher concentrations than in seed coat samples. Zhou et al. [47] isolated sciadopitysin, ginkgetin and isoginkgetin from sarcotesta. Since the exocarp also contains biflavonoids, Shen et al. [46] developed an efficient and industrially suitable protocol for the targeted isolation of high-purity bilobetin, ginkgetin and isoginkgetin from the exocarp of ginkgo, which is considered industrial waste. They used a macroporous adsorption resin to enrich the biflavonoid content and then used a two-dimensional preparative normal-phase/reversed-phase HPLC-DAC system for isolatopn. 

The presence of biflavonoids may also depend on the stage of leaf development. Wang et al. [33] reported the content of ginkgetin, isoginkgetin, bilobetin and sciadopitysin in leaves at six developmental stages. The content of biflavonoids ranged from 0 to 800 ng/g and depends significantly on the developmental stage. Sciadopitysin was present in all samples analyzed, but the amount was highest in the more advanced stages. The highest amounts in the last developmental stage were also reported for bilobetin and gingketin, while the content of isoginkgetin was the highest in the youngest leaves [33]. The age of the plant may also affect the accumulation of biflavonoids. According to Pandey et al. [44] 175-year-old trees accumulate more sciadopitysin in leaves and stem than 8-year-old trees. 

Kaur et al. [41] studied the content of bilobetin, ginkgetin and sciadopitysin by reverse-phase high-performance thin-layer chromatography (RP-HPTLC) in ginkgo leaves collected from six locations in India. They reported ginkgetin and sciadopitysin in all analyzed samples, with ginkgetin being the most abundant in five of six localities. Bilobetin was present in samples from three locations. The authors indicated that the reason for this could be the difference in growth height and that plants cultivated in fields have higher levels of bioactive constituents than naturally growing old trees.

The mechanisms of the various biflavonoid accumulations and the influence of external factors are not explained. This may be because in physiological studies involving ginkgo flavonoids, biflavonoids have often been neglected, and only data on the changes in monomeric flavonoids have been reported [49,50,51]. In articles on the influence of environmental factors such as temperature [52], light [53], UV-B radiation [54] and salt stress [55] on ginkgo flavonoids, only data for monomeric flavonoids are reported. Further research including data on biflavonoids will help to understand the physiological role of biflavonoids in ginkgo.

## 5. Biological Activity of GinkgoBiflavonoids

**Figure 3:**
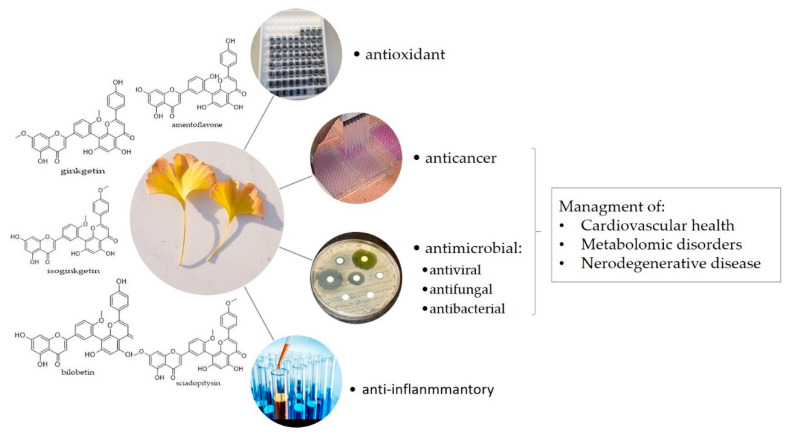
Bioactivity of *G. biloba* biflavonoids.

The biological properties and, consequently, the potential pharmacological application essentially depend on the chemical structure of flavonoids [26]. Modifications of flavonoids such as *O*-methylation, hydroxylation, glycosylation, and dimerization have a significant impact on the bioavailability and bioactivity of flavonoids and consequently on their potential role in plants and their potential health-related activities. Therefore, the biological activity of biflavonoids differs significantly from the biological activity of their monomeric structure. In the article, we have summarized the data on the biological activity of biflavonoids isolated from ginkgo in terms of their antioxidant, antimicrobial, antiviral and anti-inflammatory activities, and their potential role in management of health conditions such as neurodegenerative, cardiovascular, and metabolic diseases (Figure 3).

### 5.1. Antioxidant Activity

Ginkgo extracts are known for their antioxidant activity and their benefit in preventing diseases related to oxidative stress (reviewed by [23,56,57]). However, according to Bedir et al. [58], biflavonoids are weaker antioxidants than other compounds identified from ginkgo. The biflavonoids amentoflavone, bilobetin, ginkgetin and sciadopitysin were compared with 25 other compounds isolated from ginkgo for antioxidant activity against HL-60 (promyeoloblasts) cells [58]. Compared to other flavonoids, the biflavonoids were not very potent inhibitors of respiratory burst. Among the four biflavonoids tested, amentoflavone had the best inhibitory effect, followed by ginkgetin. Interestingly, bilobetin and sciadopitysin were the compounds with the least antioxidant activity among the 29 specialized metabolites isolated from ginkgo. 

On the other hand, amentoflavone, which was also isolated from other plant species, was reported to be a potent antioxidant [59,60], and it was suggested that dimerization, especially 3′,8″-dimerization, can increase the antioxidant capacity of flavonoids [61]. Amentoflavone showed significant antioxidant potential in vitro and in food-model systems; it exhibited strong antioxidant ability in scavenging DPPH, ABTS, superoxide, and hydroxyl radicals [59]. As summarized by Yu et al. [62] amentoflavone could be a potent molecule involved in the protection of various processes from different reactive oxygen species. Gan et al. [63] performed in vivo pharmacokinetic study on rat model to determine the plasma concentrations of amentoflavone and its metabolites. They reported that the low bioavailability of amentoflavone was due to extensive glucuronidation, but glucuronidation did not alter the antioxidant activity. 

In view of all this, the contribution of biflavonoids to the antioxidant capacity of ginkgo needs to be further investigated, especially in in vivo models because the available data are contradictory.

### 5.2. Anticancer Activity

Ginkgo leaves have shown antiproliferative and anticancer activity in several in vitro studies against different cell lines [64,65], and these effects may be related to the presence of various flavonoids which have been targets for anticancer research for a decade [66]. In recent years, biflavonoids are also more and more studied as potential antitumor agents. As summarized by Menezes and Diederich [67], the protective effects of biflavonoids on cancer might be related to the inhibition of metabolism-related processes/pathways, enzymes, or proteins, such as STAT3/SHP-1/PTEN, kinesins, tissue kallikreins, aromatase, estrogen, protein modifiers, antioxidants, autophagy, and apoptosis-induction mechanisms. 

In vitro bioassays and various cell lines are commonly used to test various natural products for their potential anticancer effects, and have also been applied to ginkgo biflavonoids. The results of in vitro studies on cell lines using ginkgo biflavonoids are summarized in Table 4. These assays are usually based on cell culture systems in which neoplastic cell lines are taken from human or other animal tumors and grown in vitro. The ability of the tested compounds to inhibit the growth (proliferation) of these cancer cells in culture is considered indicative of their potential value as cancer therapeutics in vivo. 

The best studied biflavonoids from ginkgo in terms of their anticancer potential are ginkgetin and amentoflavone. As summarized by Adnan et al. [75], ginkgetin combats cancer progression by arresting the cell cycle, inducing apoptosis, stimulating autophagy, and targeting many deregulated signaling pathways such as JAK/STAT and MAPKs. It may act as a potent Hsp90 inhibitor [76]. Heat-shock protein 90 (Hsp90) is an important molecular chaperone that is overexpressed in cancer cells and may be a therapeutic drug target for cancer chemotherapy. Ginkgetin has been shown in several studies to be a promising compound agent that enhances the anti-cancer effects of other drugs/compounds. Lou et al. [77] reported that ginkgetin isolated from ginko leaves promoted an anticancer effect induced by cisplatin and that this mechanism was via ferroptosis-mediated disruption of the Nrf2/HO-1 axis in EGFR wild-type non-small-cell lung cancer. In synergy with resveratrol ginkgetin, it can suppress vascular endothelial growth factor (VEGF) and may represent a therapeutic strategy in cancer [78]. 

According to recent data, amentoflavone is involved in anti-cancer activity by mediating various signaling pathways such as extracellular signal-regulated kinase (ERK), nuclear factor kappa-B (NF-κB) and phosphoinositide 3-kinase/protein kinase B (PI3K/Akt) (reviewed by [79]). In a comparative study of ginkgetin and sciadopitysin, Choi et al. [80] evaluated their effect on the inhibition of phosphatase in regenerating liver-3 (PRL-3), a key factor in the acquisition of metastatic properties of tumor cells. The authors showed that both compounds moderately inhibited PRL-3, but this effect was higher than in the monomeric flavonoids acacetin and apigenin.

Bilobetin, isoginkgetin and sciadopitysin have been less studied but may also be involved in the anticancer effect of ginkgo extracts. Li et al. [45] tested five biflavonoids isolated from ginkgo for their anti-proliferative activities on HepG2, HeLa, and NCI-H460 cell lines and reported that bilobetin and isoginkgetin exhibited the better anti-proliferative activities in different cancer lines. The significant morphological changes of the cells confirmed their anticancer effects in a dose-dependent manner. They were able to arrest the G2/M phase of the cell cycle, induce the apoptosis of HeLa cells in a dose-dependent manner, and activate the proapoptotic protein Bax and the executor caspase-3. Bilobetin could also inhibit the antiapoptotic protein Bcl-2 [45]. These could be the mechanisms underlying its anti-proliferation. 

Although in vitro data suggest an anticancer effects of ginkgo biflavonoids, in vivo studies had to confirm these effects. For example, pharmacokinetic studies in rats showed that the oral bioavailability of amentoflavone is quite low and in plasma glucuronidated amentoflavone is predominantly found [63]. In the same study, they reported that glucuronidated amentoflavone metabolites have lower anticancer activity than amentoflavone. Therefore, further in vivo and clinical studies need to confirm ginkgo biflavonoids as potential anticancer agents.

### 5.3. Antiviral Activity

For more than 20 years, biflavonoids have been investigated as potential antiviral agents and shown inhibitory activities against several viruses including respiratory viruses (influenza A, influenza B, respiratory syncytial, parainfluenza type 3, adenovirus type 5, measles, severe acute respiratory syndrome including SARS-CoV2), herpesviruses (HSV-1, HSV-2, HCMV, VZV, and Epstein–Barr virus (EBV)), human immunodeficiency virus (HIV), protozoal (Leishmaniasis, Malaria) and viruses which cause dengue, coxsackieviral, hepatitis, etc. [37,67,81]. In recent years, during the coronavirus pandemic, amentoflavone has become a molecule of interest due to its antiviral effects against SARS-CoV-2 based on in vitro and in silico studies [82,83]. Amentoflavone in vitro has the potential to inhibit herpes simplex virus 1 (HSV-1) including Acyclovir (ACV) (antiviral drug) -resistant strains [84]. 

The anti-influenza virus properties of ginkgetin, isolated from leaves of ginkgo, were revealed via inhibiting the activity of sialidase, a key enzyme that plays an essential role in the life cycle of the influenza virus and is a crucial drug target for the development of effective anti-influenza medications. The antiviral activity of ginkgetin was determined against two types of influenza viruses sialidases (A/PR/8/34 (H1N1) and A/Guizhou/54/89 (H3N2)) with 50% inhibitory concentration values of 55.00 and 9.78 µg/mL, respectively [85]. In another investigation, Haruyama and Nagata [86] assessed the anti-infectivity properties of ginkgo leaf extract against influenza viruses A (H1N1 and H3N2) and B (B/Lee/40), and the results revealed notable antiviral actions against the investigated viruses. The authors have suggested that ginkgetin and other biflavonoid molecules present in extracts might be accountable for the antiviral effects against influenza viruses with a proposed mechanism via repressing the function of hemagglutinin in adsorption to host cells. 

In a combined in vitro and in silico study, biflavonoids such as sciadopitysin, ginkgetin, isoginkgetin, amentoflavone, and bilobetin were examined for their anti-severe acute respiratory syndrome coronavirus 2 (anti-SARS-CoV-2) activities via targeting 3-chymotrypsin-like protease (3CLpro), an important enzyme that is required for the replication of coronaviruses, including SARS-CoV-2. The results of inhibition assay demonstrated that the tested compounds show notable anti-SARS-CoV-2 3CLpro actions. The results also showed that sciadopitysin is the most potent inhibitor of SARS-CoV-2 3CLpro among the test biflavonoids with reversible and mixed inhibition mechanisms. The mechanisms have been confirmed by inhibition kinetic studies and molecular docking simulations [87]. 

### 5.4. Anibacterial and Antifungal Activity

Extracts of ginko possess remarkably high inhibitory activity against a range of Gram-positive and Gram-negative bacteria [88], and this activity may be related to the presence of biflavonoids with antibacterial activity [19,67]. The best studied biflavonoid from ginkgo, in terms of its antimicrobial activity, is amentoflavone. Hwang et al. [89] tested the antimicrobial activity of amentoflavone against *Enterococcus faecium, Staphylococcus aureus*, *Streptococcus mutans*, *Escherichia coli* O-157, *Escherichia coli*, and *Pseudomonas aeruginosa* and showed that amentoflavone had a considerable antibacterial effect and synergistic interaction with antibiotics against various bacterial strains except *S. mutans*. Amentoflavone has confirmed antimicrobial activity against foodborne pathogens, including *S. aureus* and *E. coli*, which was also confirmed in food models using minced chicken meat and apple juice food models [59]. Due to its antimicrobial activity against the cyanobacterium *Microcystis aeruginosa* [90], it also has potential in the treatment of harmful cyanobacterial blooms. Cyanobacterial blooms are becoming a global environmental problem due to the destruction they cause in freshwater ecosystems. Amentoflavone [91] and ginkgetin [92] showed antimicrobial effects in vitro and in vivo in mouse models against *Streptococcus suis*, a zoonotic pathogen, which can cause considerable economic losses in the swine industry and severe public health issues. Bagla et al. [93] reported the antimicrobial activity of isoginkgetin against *E. faecalis*, *S. aureus*, *P. aeruginosa* and *E. coli*. The antimicrobial activity of bilobetin and sciadopitysin is poorly studied.

The biflavonoids of ginkgo are also potent antifungal agents. Krauze-Baranowskaa and Wiwart [94] tested amentoflavone, 7-*O*-methylamentoflavone (sequoiaflavone), bilobetin, ginkgetin, sciadopitysin and 2,3-dihydrosciadopitysin for their antifungal activity against *Alternaria alternata, Cladosporium oxysporum* and *Fusarium culmorum*. They reported that bilobetin exhibited significant antifungal activity and completely inhibited the growth of *C. oxysporum* and *F. culmorum*, but the activity of ginkgetin and 7-*O*-methylamentoflavone against *A. alternata* was stronger than that of bilobetin. In addition, biflavones without a methoxyl group such as amentoflavone and biflavonoid consisting of flavanone–flavone units such as 2,3-dihydrosciadopitysin were inactive or weak against *A. alternata* and *C. oxysporum*. The significant antifungal activity of biflavonoes with methoxyl group has been found mainly against *C. oxysporum* and increase in the number of methoxyl groups decreased antifungal action of biflavones against *A. alternata* [94]. Amentoflavone has been reported to be an effective molecule against the human pathogenic yeast *Candida albicans* and the antifungal mechanisms may be due to physiological changes inducing S-phase arrest in the intracellular environment [95]. Hwang et al. [96] reported that amentoflavone induces apoptosis in *C. albicans* and causes mitochondrial dysfunction. 

### 5.5. Anti-Inflammatory Effect

Biflavonoids shown anti-inflammatory activity by inhibiting phospholipase A2 or regulating pro-inflammatory gene expression in vitro and in vivo [97]. Zhou et al. [98] compared the anti-inflammatory effects of ginkgetin and isoginkgetin using placental alkaline phosphatase reporter assay, developed to measure nuclear factor kappa B. Both biflavonoids showed inhibitory effects, with the effect of ginkgetin being greater than that of isoginkgetin. Kim et al. [99] studied in vitro the effects of five flavonoids, including amentoflavone and isoginkgetin on epidermal cyclooxygenase/lipoxygenase, as their anti-inflammation potential. In their study, the amentoflavone showed potent and selective inhibitory activity on cyclooxygenase, which was comparable to those of anti-inflammatory drug indomethacin. In the same study, ginkgetin showed a weak inhibitory effect on cyclooxygenase.

Li et al. [100] isolated 18 different chemical components from the flowers of ginkgo, including the biflavonoids amentoflavone, sciadopitysin, bilobetin and isoginkgetin. They evaluated their anti-inflammatory ability in the lipopolysaccharide induced RAW264.7 macrophages. Among all tested compounds, the most effective compounds were bilobetin and isoginkgetin. They exhibited significant dose-dependent inhibitory effects on tumor necrosis factor-α IL-6, PGE2, inducible NO synthase mRNA, and cyclooxygenase-2 mRNA levels. According to the authors, they can be promising candidates for the development of new anti-inflammatory agents.

### 5.6. Potential Role in the Management of Cardiovascular and Metabolomics Diseases

Extracts from ginkgo leaves are widely used in both Eastern and Western countries for the treatment of metabolic diseases (such as hyperlipidemia), but the bioactive compounds underlying these mechanisms have not been fully characterized [101]. Liu et al. [101] investigated the inhibitory potential and mechanism of isoginkgetin, ginkgetin, bilobetin and sciadopitysin isolated from ginkgo on pancreatic lipase, a key target regulating lipid absorption. Their results clearly demonstrated that all tested biflavones displayed strong-to-moderate inhibitory effects on pancreatic lipase. Wang et al. [102] also hypothesized that biflavonoids may play a potential role in the treatment of cerebrovascular disease. They isolated bilobetin, ginkgetin, isoginkgetin and sciadopitysin from ginkgo and screened them using oleic acid-induced lipid production in HepG2 cells. They also used reverse targeting and molecular dynamics simulation for screening the non-covalent effects of biflavonoids on the potential targets of atherosclerosis. The interactions between biflavonoids and potential targets were evaluated by an exogenous cell model. Among tested biflavonoids, ginkgetin significantly inhibited oleic acid-induced lipid production and reduced total cholesterol and triglyceride levels. The authors concluded that it can be promising natural medicine for the treatment of atherosclerosis. Dell’Agli et al. [103] studied the ability of five ginkgobiflavonoids, ginkgetin, sequoiaflavone, amentoflavone, bilobetin and sciadopitysin, to inhibit cAMP phosphodiesterase activity and promote vasorelaxation. They reported that all biflavones inhibited PDE5A1 in a concentration-dependent manner, with ginkgetin being the most potent, followed by bilobetin, sciadopitysin, amentoflavone and sequoiaflavone.

The best-studied biflavonoid from ginkgo regarding cardiovascular health protection is amentoflavone and several studies in recent years have highlighted its therapeutic potential. Kubota et al. [104] reported that amentoflavone significantly increased the heartbeat rate, and it is considered one of the main components of ginkgo that produces the beneficial chronotropic and inotropic effects. Amentoflavone can ameliorate the myocardial ischemia-reperfusion injury, as shown in in vivo and in vitro studies [105]. In an in vivo study in mice suffering from metabolic syndrome amentoflavone was shown to inhibit the changes in general metabolic parameters, including body weight, fat mass, insulin levels, and glucose tolerance activity, and it may markedly protect against cardiovascular dysfunction [106]. Amentoflavone prevents oxidized low-density lipoprotein-induced lipid accumulation and as such may be involved in protection from atherosclerotic cardiovascular disease [107].

### 5.7. Potential Role in the Management Neurodegenerative Disorders Treatment

The use of ginkgo for the treatment of neurodegenerative diseases is widely recognized [15]. As described by Uddin et al. [108], biflavonoids are molecules that have the potential to noticeably alter the aggregation of tau and Aβ and efficiently reduce the toxic effect of Aβ-oligomers compared to monoflavonoids. Thus, these are potent molecules for the treatment and prevention of Alzheimer’s disease. 

Kang et al. [109] investigated the neuroprotective effects of nine naturally occurring bi-flavonoids on oxidative stress and amyloid-β-peptide-induced cell death in neuronal cells. In this study, bilobetin, ginkgetin, isoginkgetin, and sciadopitysin purified from ginkgo and amentoflavone from other source were investigated. Of the biflavonoids tested, amentoflavone, ginkgetin, and isoginkgetin showed the strongest neuroprotective effects against cytotoxic insults induced by oxidative stress and amyloid β. These results suggest their therapeutic potential against neurodegenerative diseases such as ischemic stroke and Alzheimer’s disease. Ginkgetin isolated from ginkgo showed neuroprotective effects against neurological damage in Parkinson’s disease in vivo and in vitro. The mechanism is via regulation of iron homeostasis by reducing oxidative damage, activating microglia, and enhancing neurotrophic potential [110,111]. As summarized by Adnan et al. [75], ginkgetin shows potent neuroprotective effects against oxidative stress-induced cell death, inhibits cerebral microbleeds, reduces neurological deficits, and stops apoptosis of neurons. Isoginkgetin also shows promising results in in vivo studies in rats as a novel therapeutic agent in the treatment of ischemic stroke injury [112]. As summarized by Varshney et al. [113], amentoflavone also has therapeutic and medicinal potential in the treatment of neurodegenerative diseases such as Alzheimer’s and Parkinson’s disease, as well as in the treatment of depression and in the prevention of aging. However, amentoflavone shows poor brain permeability in mice in vivo [114]. After treatment with ginkgo extracts, the concentration of the active compounds in the brain was below the limit of quantification. There is also some evidence that amentoflavone may be useful in the treatment of psychiatric disorders [115].

Wang et al. [116] used network pharmacology to explore the potential mechanism of ginkgo in the treatment of neurodegenerative disorders. They reported that twenty-seven ginkgo constituents can affect biological processes such as oxidation reactions and activate the activities of transcription factors. However, they did not identify any of the biflavonoids as effective molecules for the treatment of neurodegenerative disorders.

## 6. Toxicity and Safety of Ginkgo Biflavonoids

Although herbal medicines are considered relatively safe, many herbal supplements have been reported to pose potential health risks, but because the complex chemical nature of supplements makes it difficult to evaluate their efficacy and safety. Mei et al. [117] summarized the data on the potential toxicology of ginkgo from experimental studies to human case reports and showed that the currently published data support both beneficial and adverse effects of ginkgo extracts and that it depends critically on which specific extract is used. Accordingly, the constituents of ginkgo extract that may be responsible for the toxicity, genotoxicity, and carcinogenicity of ginkgo have not yet been definitively identified. 

The potential toxicity of biflavonoids from ginkgo has not been well studied. This may be because biflavonoids have only recently begun to attract greater scientific attention as potent bioactive compounds. Cardoso et al. [118] reported the mutagenic activity of amentoflavone in the *Salmonella typhimurium* assay. Amentoflavone and sciadopitysin could inhibit the activity of human UDP-glucuronosyltransferase (UGT), which is the most important class of detoxification enzymes and is highly expressed in metabolic organs such as liver, intestine, and kidney [119,120], but further studies are needed to explain this mechanism. Li et al. [121] investigated the toxicological effects of biflavonoids from ginkgo in an in vitro and in vivo study. Their results suggest that the investigated biflavonoids from ginkgo may have potential liver and kidney toxicity, so further studies on the safety and efficacy of biflavonoids from ginkgo are needed.

## 7. Conclusions and Further Directions

Ginkgo is a widely recognized medicinal plant and a natural reservoir of bioactive compounds. According to the available data, dimeric flavonoids—biflavonoids—are an important component of the bioactivity of ginkgo. So far, 13 biflavonoids have been identified in ginkgo, of which ginkgetin, isoginkgetin, amentoflavone, bilobetin, and sciadopitysin have been described most frequently. Most of the available work used only targeted analyses to determine biflavonoids in ginkgo, so further untargeted analyzes are needed to possibly identify additional biflavonoids structures. Their presence and content may be influenced by the growth stage, growth conditions, and age of the trees, and the accumulation of compounds in different parts of the plant varies. So far, leaves have mainly been studied, but biflavonoids are also detected in other plant parts such as stem, flowers and various seed parts. According to the available data, among the identified biflavonoids, sciadopitysin is often reported as one with the highest content. As far as we know, the role of biflavonoids in ginkgo plants is not known and further research in this direction would be useful.

The potential health benefits of biflavonoids from ginkgo are better understood. As we have summarized, the available data suggest their antioxidant, anticancer, antiviral, antibacterial, antifungal, and anti-inflammatory effects, as well as a possible role in the treatment of cardiovascular, metabolic, and neurodegenerative diseases. The best studied biflavonoid from ginkgo is amentoflavone, but according to the literature, other biflavonoids from ginkgo may also be potent pharmacological compounds. It is important to note that toxicological studies have also shown potential toxicity of ginkgo biflavonoids. Therefore, further research should consider these effects when evaluating their bioactive properties. The active concentration and safe range for ingestion should be determined.

In summary, biflavonoids play an important role in the biological activity of ginkgo, but there is still a lot to be clarified. They are also of interest from a plant biology/physiology point of view as they could play an important role in plant–environment interactions, and research in this area is needed due to the current problems with climate change. On the other hand, they are molecules that have the potential to be used in the pharmaceutical industry. However, most studies are conducted in vitro, and further in vivo and clinical studies should determine their efficacy and safety.

## Figures and Tables

**Table 1:** Chemical structures of the flavone-flavone biflavonoids in ginkgo.

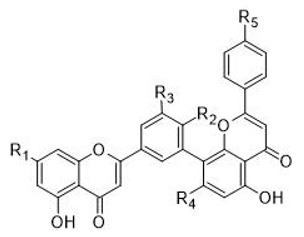					
	R_1_	R_2_	R_3_	R_4_	R_5_
**1** amentoflavone	OH	OH	H	OH	OH
**2** sequoiaflavone	OCH_3_	OH	H	OH	OH
**3** bilobetin	OH	OCH_3_	H	OH	OH
**4** podocarpusflavone A	OH	OH	H	OH	OCH_3_
**5** ginkgetin	OCH_3_	OCH_3_	H	OH	OH
**6** isoginkgetin	OH	OCH_3_	H	OH	OCH_3_
**7** 5′-methoxybilobetin	OH	OCH_3_	OCH_3_	OH	OH
**8** sciadopitysin	OCH_3_	OCH_3_	H	OH	OCH_3_
**9** 7″-*O*-β-d-glucosyl-ginkgetin	OCH_3_	OCH_3_	H	O-Glc	OH
**10** 7″-*O*-β-d-Glucosyl-isoginkgetin	OH	OCH_3_	H	O-Glc	OCH_3_
**11** amentoflavone 7″-*O*-d-glucopyranoside	OH	OH	H	O-Glc	OH

**Table 2:** The structure of flavanone-flavone type of biflavonoide in ginkgo.

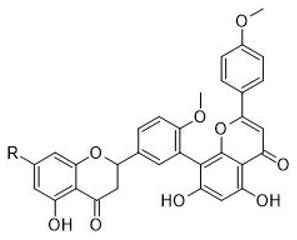	
	R
**12** 2,3-Dihydroisoginkgetin	OH
**13** 2,3-Dihydrosciadopitysin	OCH_3_

**Table 3:** Example of extraction and identification methods used for analysis of ginkgo biflavonoids from different plant parts.

Plant Part		Biflavonoids	Extraction	Identification
leaves		bilobetin, ginkgetin, isoginkgetin, sciadopitysin and dihyrosciadopitysin [38]	acetone	NMR
		amentoflavone, bilobetin, ginkgetin, isoginkgetin, scyadopytisin [39]	60% methanol	HPLC-DAD-MS
		amentoflavone, bilobetin, ginkgetin/isoginkgetin, sciadopitysin [40]	methanol	HPLC- FTICR- MS
		bilobetin, gingketin sciadopitysin [41]	Soxhlet apparatus with 70% methanol	RP-HPTLC
		bilobetin, gingketin, isoginkgetin, sciadopitysin [33]	ultrasonic extraction using 50% aqueous ethanol	HPLC-MS/MS
		bilobetin, ginkgetin, isoginkgetin, sciadopitysin [42]	MeOH-based deep eutectic solvent micellar system	UHPLC-QQQ-MS/MS
		bilobetin, gingketin, isoginkgetin, sciadopitysin [43]	ultrasonic-assisted ionic liquid extraction	HPLC-DAD NMR
		amentoflavone, sciadoptysin [44]	ethanol	UHPLC–ESI-MS/MS
stem		amentoflavone, sciadoptysin [44]	ethanol	UHPLC–ESI-MS/MS
flowers	male	amentoflavone, bilobetin, isoginketin sciadopitysin [45]	70% EtOH followed by silica gel column chromatography	NMR
fruit		amentoflavone, sciadoptysin [44]	ethanol	UHPLC–ESI-MS/MS
	exocarp	Bilobetin, ginkgetin Isoginkgetin [46]	macroporous adsorption resin and then two-dimensional preparative HPLC-DAD system	HPLC-DAD
	sarcotesta	ginkgetin, isoginkgetin, sciadopitysin [47]	petroleum ether extract	HPLC-MS/MS NMR
	seed coats	Isoginkgetin [48]	70% ethanol	UPLC-ESI-MS

**Table 4:** Results of in vitro studies on cell lines using ginkgo biflavonoids.

Testet Ginkgo Biflavonoids	Used Cell Line (s)	Main Findings
7′′-*O*-β-d-glucopyranoside bilobetin isoginkgetin sciadopitysin [45]	hepatocellular carcinoma, HepG2, cervical cancer cells, HeLa non-small-cell lung cancer, NCI-H460	bilobetin and isoginkgetin exhibited better anti-proliferative activities on different cancer lines.
ginkgetin [68]	breast cancer cells, MCF-7, T-47D, and MDA-MB	ginkgetin induces breast cancer cells with estrogen receptors via the inhibition of their expression
ginkgetin [69]	human prostate cancer, PC-3 cells.	ginkgetin induces apoptosis in PC-3 cells via activation of caspase 3 and inhibition of survival genes
ginkgetin [70]	human hepatocellular carcinoma, HepG2	ginkgetin significantly reduced HepG2 cell viability in a dose-dependent manner and could be a cell apoptosis stimulator by affecting the balance between cell proliferation and apoptosis
ginkgetin [71]	human immortalised myelogenous leukemia, K562	ginkgetin effectively inhibits K562 cell proliferation, and TNF-α plays a key role in ginkgetin-induced cell apoptosis
amentoflavone [72]	human bladder cancer cell line, TSGH8301	amentoflavone induces apoptosis and reduces expression of anti-apoptotic and metastasis-associated proteins
amentoflavone [73]	human ovarian cancer, SKOV3 and OVCAR-3	amentoflavone significantly suppress cell proliferation, induce apoptosis and block cell cycle progression
amentoflavone [74],	human cervical cancer, SiHa and CaSki	amentoflavone activates PPARγ/PTEN expressions and induces apoptosis via suppressing E7 expression, cell cycle arrest at sub-G1 phase, and mitochondria-emanated intrinsic pathways

## Data Availability

Not applicable.
